# Diabetes Drug Discovery: hIAPP_1–37_ Polymorphic Amyloid Structures as Novel Therapeutic Targets

**DOI:** 10.3390/molecules23030686

**Published:** 2018-03-19

**Authors:** Isaac Fernández-Gómez, Marquiza Sablón-Carrazana, Alberto Bencomo-Martínez, Guadalupe Domínguez, Reyna Lara-Martínez, Nelly F. Altamirano-Bustamante, Luis Felipe Jiménez-García, Karina Pasten-Hidalgo, Rosa Angélica Castillo-Rodríguez, Perla Altamirano, Suchitil Rivera Marrero, Cristina Revilla-Monsalve, Peter Valdés-Sosa, Fabio Salamanca-Gómez, Eulalia Garrido-Magaña, Chryslaine Rodríguez-Tanty, Myriam M. Altamirano-Bustamante

**Affiliations:** 1 Unidad de Investigación en Enfermedades Metabólicas, Centro Médico Nacional Siglo XXI, Instituto Mexicano del Seguro Social, Ciudad de México 06720, Mexico; isaacfg1@gmail.com (I.F.-G.); cristina_revilla@hotmail.com (C.R.-M.); 2 Departamento de Neuroquímica, Centro de Neurociencias de Cuba, Habana 11600, Cuba; marquiza09@gmail.com (M.S.-C.); bencomom1985albe@gmail.com (A.B.-M.); suchitil@cneuro.edu.cu (S.R.M); peter@cneuro.edu.cu (P.V.-S.); 3 Instituto de Fisiología Celular, UNAM, Ciudad de México 04510, Mexico; gdoming@ifc.unam.mx; 4 Departamento de Biología Celular, Facultad de Ciencias, UNAM, Ciudad de México 04510, Mexico; rlm@ciencias.unam.mx (R.L.-M.); lfjimenezgarcia@gmail.com (L.F.J.-G.); 5 Instituto Nacional de Pediatría, Ciudad de México 04530, Mexico; nellyab34@gmail.com (N.F.A.-B.); kapastencell@hotmail.com (K.P.-H.); asor108@gmail.com (R.A.C.-R.); 6 Cátedras Conacyt, Instituto Nacional de Pediatría, Ciudad de México 04530, Mexico; 7 Servicio de Medicina Nuclear, Hospital de Especialidades, CMN, La Raza, Instituto Mexicano del Seguro Social, Ciudad de México 06720, Mexico; mmab02@hotmail.com; 8 Coordinación de Investigación en Salud, Instituto Mexicano del Seguro Social, Ciudad de México 06720, Mexico; fabio.salamanca@imss.gob.mx; 9 UMAE Hospital de Pediatría, Centro Médico Nacional Siglo XXI, Instituto Mexicano del Seguro Social, Ciudad de México 06720, Mexico; garridolulu@hotmail.com

**Keywords:** IAPP, diabetes mellitus, pharmacological chaperones, amyloid structures, conformational diseases, drug discovery

## Abstract

Human islet amyloid peptide (hIAPP_1–37_) aggregation is an early step in Diabetes Mellitus. We aimed to evaluate a family of pharmaco-chaperones to act as modulators that provide dynamic interventions and the multi-target capacity (native state, cytotoxic oligomers, protofilaments and fibrils of hIAPP_1–37_) required to meet the treatment challenges of diabetes. We used a cross-functional approach that combines in silico and in vitro biochemical and biophysical methods to study the hIAPP_1–37_ aggregation-oligomerization process as to reveal novel potential anti-diabetic drugs. The family of pharmaco-chaperones are modulators of the oligomerization and fibre formation of hIAPP_1–37_. When they interact with the amino acid in the amyloid-like steric zipper zone, they inhibit and/or delay the aggregation-oligomerization pathway by binding and stabilizing several amyloid structures of hIAPP_1–37_. Moreover, they can protect cerebellar granule cells (CGC) from the cytotoxicity produced by the hIAPP_1–37_ oligomers. The modulation of proteostasis by the family of pharmaco-chaperones **A**–**F** is a promising potential approach to limit the onset and progression of diabetes and its comorbidities.

## 1. Introduction

Up to relatively recent data, proteins were thought of as perfectly functional and well folded structures. Nowadays, we know that they can also present disordered elements both in vitro and in vivo. Moreover, proteins tend to unfold, misfold and aggregate due to intrinsic and extrinsic causes [1,2,3]. Unfolding and aggregation of proteins gives way to what is known as “conformational diseases (CDs)”: One of the most challenging CDs is diabetes [1,4,5,6,7,8]. The prevalence of diabetes is increasing rapidly: 29.1 million people or 9.3% of the population of the USA has diabetes resulting in a total cost of $245 billion [9]. Type 2 diabetes mellitus (T2D) rates have been rising faster than type 1 diabetes mellitus (T1D), and with the increased obesity, we are currently facing a T2D pandemic in paediatric stages, thus, representing a great challenge for the medical community of the 21st century [10].

The human islet amyloid polypeptide (hIAPP_1–37_) is a 37-residue peptide produced by the pancreatic β-cell. It participates in glucose homeostasis, controlling gastric emptying, and suppression of glucagon release. The hIAPP_1–37_ deposition occurs in ∼90% of autopsies of individuals with T2D [11,12]. hIAPP_1–37_ has a propensity to aggregate with the formation of toxic oligomers and fibres that contribute to the onset of T2D [13,14]. The islet in T2D is characterized by an approximate 60% β-cell deficit, increased β-cell apoptosis, and high auto-phagosome formation [15]. The membrane-disrupting oligomers of amyloidogenic hIAPP_1–37_ initiate apoptosis, at least in part, by inducing ER stress and disrupting the proteasome [14,16,17,18,19,20,21]

Chaperones are a group of molecules that assist in the folding process of proteins. Chaperones bind to unfolded intermediaries and prevent them from interacting with other compounds. They also promote their correct folding and can rescue their functionality if they are misfolded [1,22,23,24]. It is to be noted that there are many different kinds of compounds which have been considered as chaperones [22,24], and have been classified as molecular, chemical and pharmacological [25,26,27]. The pharmaco-chaperone family can do different things: either accelerate or inhibit the protein-aggregation process (depending on their concentration levels); stabilize the native conformer; stabilize fibres; and even binding to oligomers thus, accelerating the formation of fibres [28].

Different studies have demonstrated that there are several types of compounds that can inhibit protein aggregation into amyloid structures, among these it is possible to find many polycyclic and polyphenolic substances [29,30,31,32]. All of these drugs contain aromatic rings in their general structure; various investigations based on these chemical structures aim to find molecules that can be used in treatment of CDs.

Our group described some naphthalene derivatives that can successfully bind to amyloid fibrils by forming aromatic interactions within the steric zippers’ dry surface, and disrupt their structure [28]. The pharmaco-chaperone family (Table 1) encompassed *N*-(2-aminoethyl)-*N*′-1-naphthylsuccinamide **A**; methyl (2-{[4-(1-naphthylamino)-4-oxobutanoyl]amino}ethyl) dithiocarbamate **B**; (2*R*)-2-(6-methoxy-2-naphthyl)propanoic acid (*Naproxen*) **C**; *N*-[4-(1-naphthylamino)-4-oxobutanoyl]-β-alanine **D**; 6-{[4-(1-naphthylamino)-4-oxobutanoyl]amino} hexanoic acid **E**; *N*3,*N*3′-ethane-1,2-dyilbis(*N*1-1-naphthylsuccinamide) **F**; and *N*-(4-aminobutyl)-*N*′-1-naphthylsuccinamide **G**. All of them have chemical structures related to naproxen (**C**)—whose anti-aggregating property against the β-fibril conformation has been previously demonstrated—and are a potential chemical scaffold to generate novel and promising antiamyloidogenic agents [33,34,35].

According to Sablón [28], it was noteworthy for us to extend this evaluation to hIAPP_1–37_ as a target protein since it provides a reasonable foundation for treating T2D. In the present study a pharmacophore-based virtual screening is applied (using the pharmaco-chaperone family above mentioned), alongside cross-functional experimental methodologies in order to study its effect on protein aggregation and fibril formation process of hIAPP_1–37_; thus, shedding light upon the discovery of novel therapeutic approaches in T2D. The discovery of pharmaco-chaperones family as potent modulator with multi-target capacity in the hIAPP_1–37_ aggregation-oligomerization process, supports the idea that it may be a fruitful approach to delay the onset and progression of diabetes.

## 2. Results

### 2.1. Molecular Framework of Interaction of Pharmacological Chaperones and the Human Islet Amyloid Polypeptide

hIAPP_1–37_ has been implicated in the pathology of type 2 diabetes mellitus: in 90% of *post-mortem* studies amyloid plaques were found in the patient’s pancreas. Hence, this protein is a target to find breaking agents of the β conformer. Eisenberg et al. [36] described that the IAPP molecules are capable of adopting a dimeric structure and that this structure is an intermediate on the pathway to fibrillation. Additionally, in the study, they suggest that the non-fibrillary state of hIAPP_1–37_ can form dimers with the two IAPP molecules interacting at helical interfaces centred at the aromatic stack of two Phe15 residue [36]. In others reports, Eisenberg et al. studied the influence of different sequence fragments of amino acid (a.a) on the conformational polymorphism of the hIAPP_1–37_ peptide [37]. Because of this, the A_13_NFLVH_18_, L_16_VHSSN_21_, F_23_GAILSS_29_ and N_22_FGAILS_28_ sequences—which are located in the molecular zipper of the IAPP conformation—were specifically studied. In addition, the contribution of His and Asp amino acids in the formation of these zones were evaluated. According to their in silico findings, these sequences have a high propensity of the fibril core of IAPP to form anti-parallel β sheet structures over parallel structures.

In our work, molecular docking studies were carried out with the 2L86—a micelle-stabilized NMR structure—which is a structure in non-amyloid form of hIAPP_1–37_. This type of molecule is used as reference in the existing literature and—in studies that regard the fibrillation process—is generally considered as a native structure of the hIAPP_1–37_ since it does not form any amyloid structures [28,38,39,40,41,42]. For example, in the correlation studies between structure and function of the hIAPP, the two structures of hIAPP_1–37_ that are used for docking studies and MD simulation, are the NMR structures of hIAPP_1–37_ bound to SDS-micelles (PDB 2L86 or 2KB8). This is the initial monomeric protein structure for investigating the interaction of small molecules [38,39]. During an investigation Nedumpully-Govindan et al. applied atomistic DMD simulations to uncover the anti-aggregation mechanism of two polyphenol molecules (curcumin and resveratrol) using as initial structure PDB 2L86 [43]. In a similar investigation, Lolicato et al. demonstrated that the resveratrol interferes with the aggregation of membrane-bound human-IAPP by an extensive set of MD simulations—the initial protein structure they used was PDB 2KB8 [44]. In another investigation Misra et al. performed molecular docking of dehydrophenylalanine (DF) that contained peptides with the 3D structure of hIAPP (2kB8), and demonstrated the inhibition of hIAPP fibrillization using the initial structure PDB 2KB8 [45].

**Figure 1:**
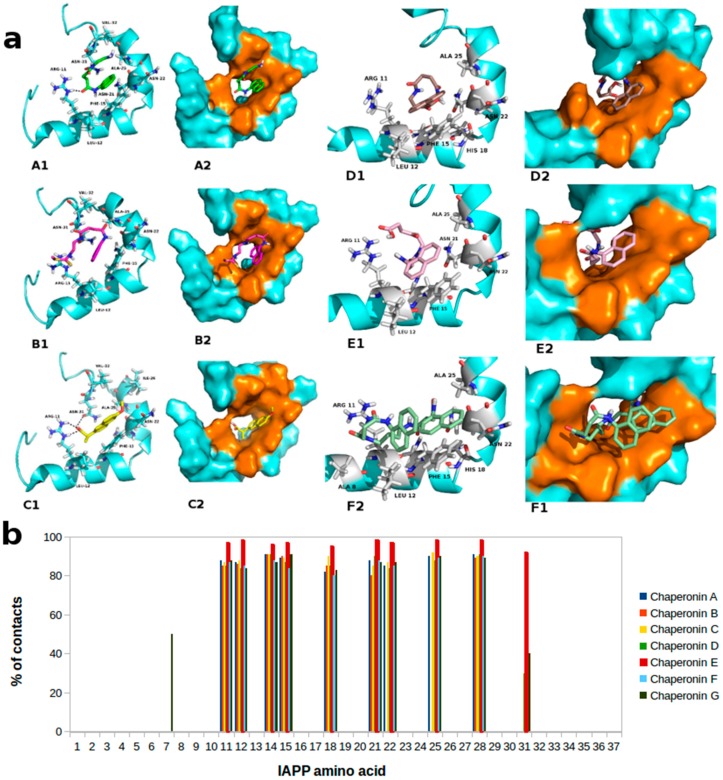
Molecular docked model of most probable interaction of pharmaco-chaperones located within the pseudo-cavity of hIAPP_1–37_ in cartoon and sphere views. (**a**) At 5 Å distance the amino acids residues surrounding chaperones (**A**, **B**, **C**, **D**, **E** and **F**) are represented in orange colour. The H-bonding interaction between chaperones and amino acids residues of hIAPP_1–37_ are shown as a dotted line; (**b**) Most probable docking interaction of chaperones with the hIAPP_1–37_ amino acid sequence.

The aim of this study was to identify which amino acids of hIAPP_1–37_ peptide interact with the evaluated pharmacological chaperones and to verify if they are in agreement with ones involved in the formation of the zipper structure of fibrils. To do this, the conformer with the lowest binding energy was calculated from 10 different conformations for each docking simulation at 5 Å distance between the chaperones and hIAPP_1–37_. This permitted us to locate all potential docking sites of chaperones on the hIAPP_1–37_ structure. The location of the sites in the 3D structure of hIAPP_1–37_ are shown in Figure 1.

In all docking assays the chaperones interact in the same pseudo-cavity formed in the helical hIAPP_1–37_. These results revealed that the chaperones bind to hIAPP_1–37_ region essentially via hydrogen bonds, hydrophobic interactions and by Van der Waals forces, mainly with the Arg11, Leu12, Asn14, Phe15, His18, Asn21, Asn22, Ala25 and Ser28 residues. The interactions π∙∙∙π were located between naphthyl ring of each chaperones and phenyl ring of the Phe15. In addition, the hIAPP_1–37_ amino acid alignment, as percent of with a.a., was explored to determine the specific interaction between them and the chaperones (Table 2).

**Figure 2:**
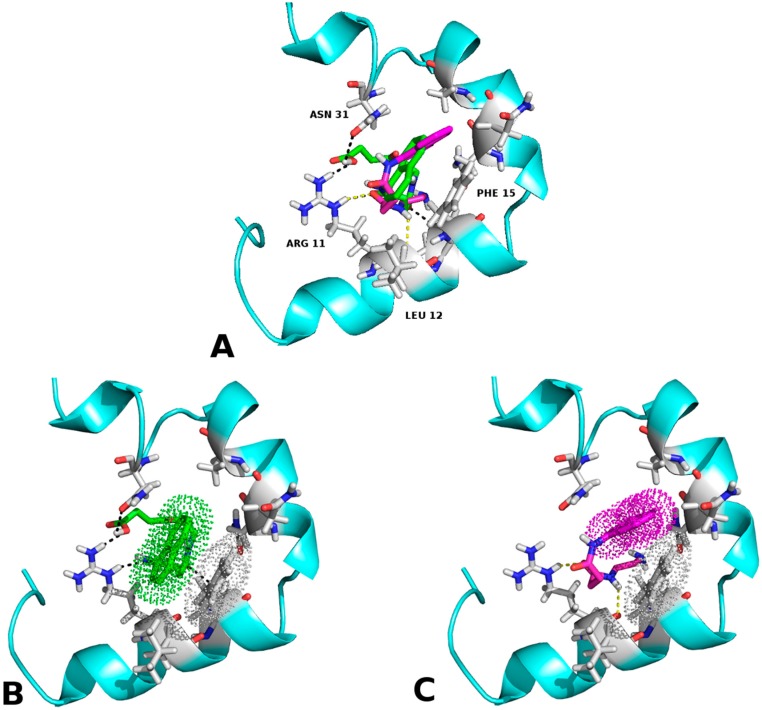
(**A**) Comparative representation of the interaction between chaperone **A** (magenta) and **E** (green). The hydrogen bonds formed by chaperone **E** are illustrated in black while the hydrogen bonds formed by chaperone **A** are depicted in yellow; (**B**) Hydrophobic interaction between amino acid Phe 15 and chaperone **E**—it is to be noted that the orientation of the electronic “clouds” are coupled—; (**C**) Hydrophobic interaction between amino acid Phe 15 and chaperone **A**. Contrary to (**B**), the orientation of the electronic “clouds” do not match.

In all docking studies carried out hydrogen bonds (H-bonds) were found between the hydrogen atom of amino group of the Arg11 and the oxygen atom of the carbonyl moiety of amide, carboxylic and succinimidyl groups of the molecule. The chaperone **E**, unlike of the rest of chaperones, has four hydrogen-bonding interactions. In particular, a double H-bond with the Arg11 was established between the oxygen atoms of the terminal carboxyl group and the carbonyl moiety (close to naphthyl ring) of chaperone **E** with the hydrogen atoms of the primary and secondary amine groups of Arg11, respectively. In addition, another H-bond was located between the hydrogen atoms of amino group of the Asn14 with the oxygen atom of the amide-group of the succinimidyl chain of chaperone **E**. The last one was found between the hydrogen atoms of the terminal carboxyl group with the oxygen atom of the amide group of the Asn31 (Figure 2). The possibility of forming multiple H-bonds in chaperone **E** could be explained by its length of amidoalkylic chain, which allows it a higher conformational flexibility.

### 2.2. Molecular Framework of Interaction of Pharmacological Chaperones ***A*** and ***E*** with the Human Islet Amyloid Polypeptide Fibrillar Structure

The hIAPP_1–37_ amyloid fibrils are very stable protein assemblies; they are built on steric zipper spines, resist SDS treatment and withstand in the pancreatic tissue [21,46,47,48,49].

Great efforts have been made in the last decade to inhibit the production of amyloid fibres, limit their growth and propagation, and diminish its fibre formations. Our approach to identify the best chaperone that avoids growth and propagation of the fibres, is capping fibrils with docking chaperones. To do this we rendered a docking with amylin fibres with the chaperones **A** and **E**: The first one has a mild effect, while the second one inhibits the formation, growth and propagation more effectively [40,41,47,50].

**Figure 3:**
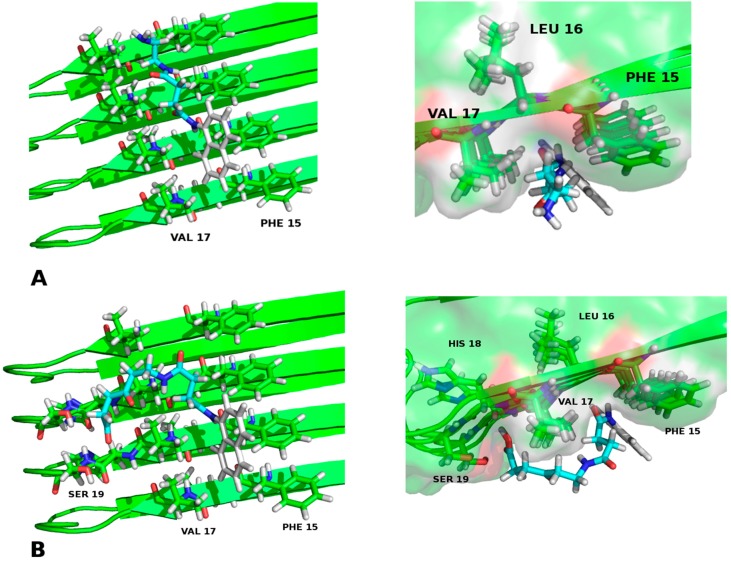
(**A**) Chaperone **A**–Amylin fibrils (4 monomers) interaction; (**B**) Chaperone **E**–Amylin fibrils (4 monomers) interaction. As shown, there is a hydrophobic interaction between phenylalanine 15, valine 17 and the naphthyl group of each chaperone. Additionally, there is a hydrogen bond between serotonin 19 and chaperone **E**.

With Figure 3 we demonstrated that in this arrangement—which resulted more proficient—a hydrophobic interaction could be established between the side chains of phenylalanine 15, valine 17 and the naphthyl group of each ligand, ergo the closeness of the electronic “clouds” π in the space (similar results were obtained for the RMN conformations of the hIAPP). Furthermore, the existence of hydrogen bonds between chaperone **E** and the fibres with serine 19 was notable. This may increase the stability of the interaction with the ligand–protein compound that was formed—thing that matches the experimental results of this work—.

**Figure 4:**
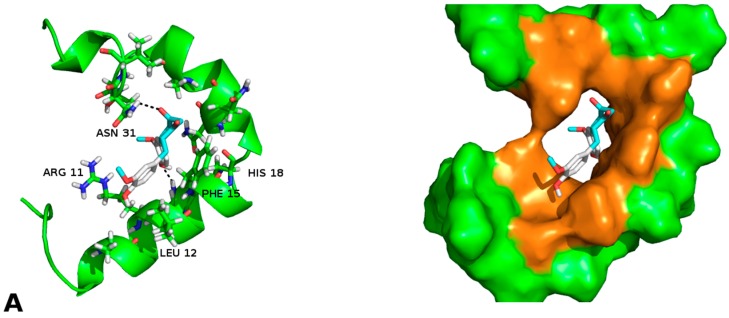

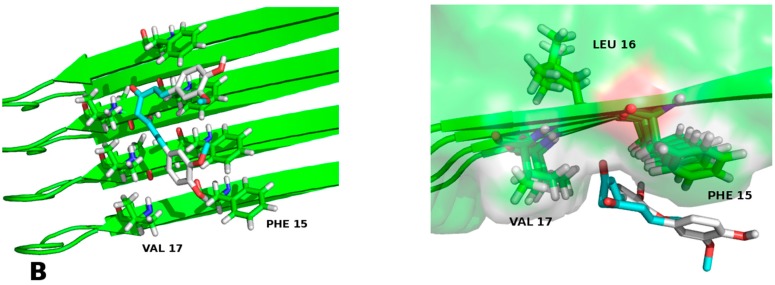
(**A**) Curcumin–hIAPP_1–37_ interaction; (**B**) Curcumin–Amylin fibrils (4 monomers) interaction. The similitude of the amino acids that participate in the protein-ligand interaction is to be noted. They are the same as those in the interaction of the pharmaco-chaperone **A** and **E**.

Curcumin was used as a positive control in the docking studies since other studies showed that: (a) it binds fibres; and (b) it affects the fibrillation process and its propagation [6,46,50,51,52]. The interaction between curcumin, the native structure and the amylin fibres, is shown in Figure 4.

### 2.3. hIAPP_1–37_ Misfolding: Aggregation and Amyloid Formation of Toxic Species and Its Modulations by the Family of Pharmaco-Chaperones

**Figure 5:**
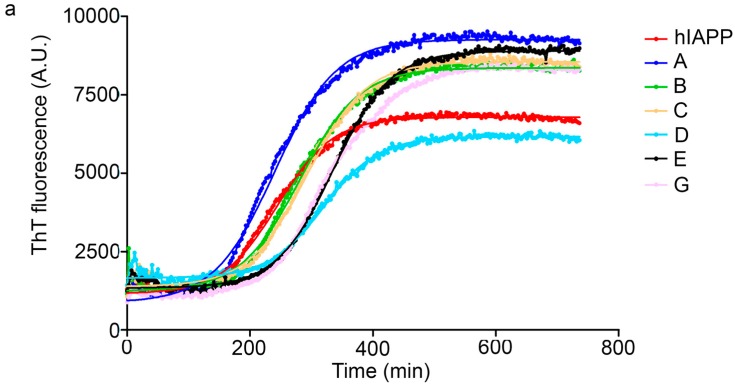

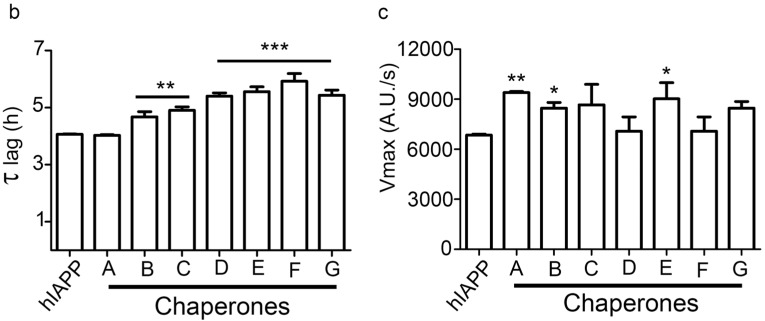
Kinetics, ν max and τ lag in hIAPP_1–37_ aggregation incubated with the chaperones **A**, **B**, **C**, **D**, **E**, and **G** at 37 °C. All data corresponds to equimolar relation between hIAPP_1–37_ and each pharmaco-chaperone (12.8 µmol/L). (**a**) The kinetics adjust to a sigmoidal curve. hIAPP_1–37_ only = red line, chaperone **A** = blue line, chaperone **B** = green line, chaperon **C** = orange line, chaperone **D** turquoise line, chaperon **E** = black line, Chaperon **G** = pink line. Chaperone **F** not shown; (**b**) Phase lag in hIAPP_1–37_ aggregation and fibril formation reached by the chaperones **A**, **B**, **C**, **D**, **E** and **G**. All chaperones reveal a superior time compared to hIAPP_1–37_, except chaperone **A**; (**c**) Vmax in hIAPP_1–37_ aggregation and fibril formation were reached by the chaperones **A**, **B**, **C**, **D**, **E** and **G**. * p < 0.05, ** p < 0.01, *** p < 0.001 when compared with hIAPP_1–37_ condition only as control. One-way ANOVA and *post-hoc* Newman–Keuls Multiple Comparison Test.

**Figure 6:**
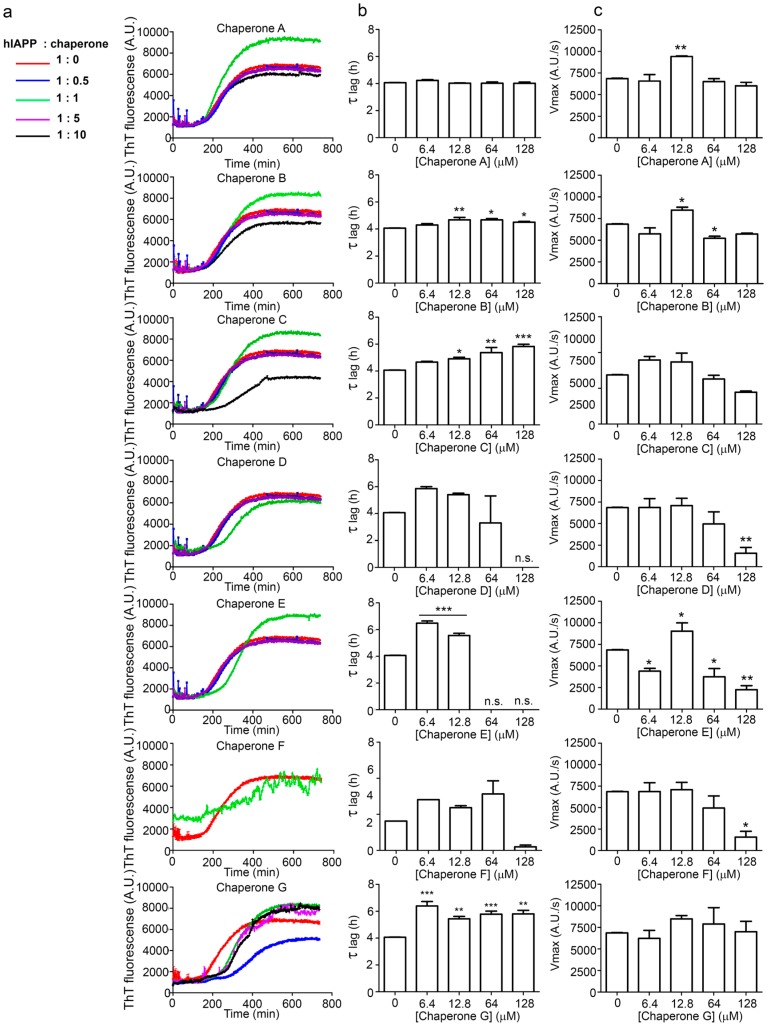
Kinetics, ν max and τ lag in hIAPP_1–37_ aggregation with the chaperones **A**, **B**, **C**, **D**, **E**, **F** and **G**. The experiments were conducted in 20 mM Tris-HCl, 100 mM NaCl (pH 7.5) at 37 °C. In all the conditions the chaperones were incubated with hIAPP_1–37_ (12.8 µmol/L). (**a**) We incubated the chaperones at different molar relations. Blue line 1:05 (12.8 µmol/L:6.4 µmol/L), green line 1:1 (12.8 µmol/L:12.8 µmol/L), purple line (12.8 µmol/L:64 µmol/L), and black line (12.8 µmol/L:128 µmol/L), using the oligomers of hIAPP_1–37_ (red line) as the control (12.8µmol/L); (**b**) Phase lag (τ lag) in hIAPP_1–37_ aggregation and fibril formation reached by the chaperones **A**, **B**, **C**, **D**, **E**, **F** and **G**; (**c**) Vmax in hIAPP_1–37_ aggregation and fibril formation reached by the chaperones **A**, **B**, **C**, **D**, **E**, **F** and **G**. * p < 0.05, ** p < 0.01, *** p < 0.001 when compared with hIAPP_1–37_ condition only as control. One-way ANOVA and *post-hoc* Newman–Keuls Multiple Comparison Test. n.s. = data not shown.

An augmentation in the intensity of the ThT fluorescence in time was observed in the sigmoidal curves, this shows an increase in the formation of fibrils from hIAPP_1–37_*.* hIAPP_1–37_ was incubated at a concentration of 12.8 µmol/L to develop the kinetics of aggregation. Then, hIAPP_1–37_ was incubated with each chaperone in a molar ratio of 1:0.5 (6.4 µmol/L), 1:1 (12.8 µmol/L), and 1:1.5 (19.2 µmol/L) (Table 1, Figure 5 and Figure 6). Considering the equimolar conditions, chaperone **A** showed a lag phase similar to the hIAPP_1–37_ alone (Figure 5a,b). At the final time, a significant increase was observed in the ThT fluorescence intensity (9509 ± 1032 A.U./s) compared with hIAPP_1–37_ alone (6944 ± 772 A.U./s), which was 37% superior. (Figure 5b,c). This suggests that chaperone **A** facilitates fibril formation.

Chaperons **B**, **C**, **D**, **E**, **F** and **G** showed a significant increase in the lag phase compared to hIAPP_1–37_ alone (3.989 ± 0.373 h), and at equimolar concentrations (Figure 5 and Figure 6, Table 1), suggesting a delay in the process of aggregation.

Chaperones **B** and **E** revealed a similar kinetic profile. These chaperones had a significantly higher Vmax (B = 8562 ± 110 A.U./s and E = 9055 ± 149 A.U./s) compared with hIAPP_1–37_ (6944 ± 772 A.U./s) (Figure 5a,c), but in a larger dose of 64 µmol/L it decreased (B = 5328 ± 672 A.U./s and E = 4465 ± 835 A.U./s) (Figure 6 and Table 1). This suggests that both chaperones delay the aggregation of hIAPP_1–37_, but could also avoid fibril aggregation (larger dose).

Chaperones **C**, **D**, **F** and **G** did not show significant differences in the Vmax with respect to hIAPP_1–37_ alone (Figure 5a,c).

Chaperone **D** showed an effect of dose-response, when increasing its concentration, the intensity of the fluorescence decreases, which is proportional to the number of fibrils formed (6.4 µmol/L = 6946 ± 1126, 12.8 µmol/L = 6326 ± 944 and 64 µmol/L = 5255 ± 289) (Figure 6 and Table 1). This suggests that chaperone **D** avoids the aggregation of hIAPP_1–37_ to fibrils.

**Figure 7:**
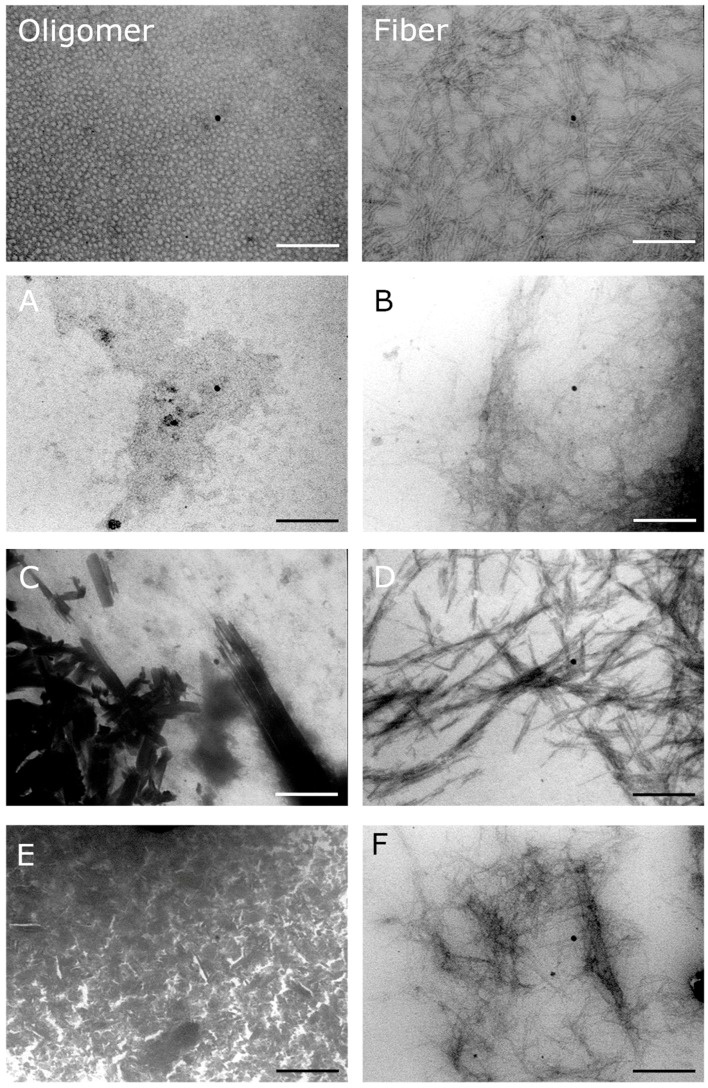
Morphological analysis of hIAPP_1–37_ aggregation process with and without chaperones (**A**–**F**) by TEM. The image Oligomer and fibre are the control with hIAPP_1–37_ alone. The electron micrographs show the aggregation process of hIAPP_1–37_ with or without incubation with chaperones in different relations. All conditions had hIAPP_1–37_ chaperone (12.8 µmol/L). (**A**) hIAPP_1–37_ vs. Chaperone **A** 1:1, there is presence of big oligomers and some fibrils; (**B**) hIAPP_1–37_ vs. Chaperone **B** 1:5, fibril aggregation; (**C**) hIAPP_1–37_ vs. Chaperone **C** 1-1, no presence of cytotoxic oligomers; (**D**) hIAPP_1–37_ vs. Chaperone **D** 1-1, thick fibrils were aggregated; (**E**) hIAPP_1–37_ vs. Chaperone **E** 1-0.5, there were no fibril aggregation; (**F**) hIAPP_1–37_ vs. Chaperone **F** 1-1, thin fibril aggregation is evident. Scale bar = 200 nm.

Some chaperones delayed the process of fibrillation of the IAPP, thus avoiding the toxic effect of the oligomers during a time interval. In addition, some of them produced a regression from cytotoxic oligomers to oligomers or monomers depending on the dose (Figure 3 and Figure 4). All the samples in presence of chaperones were analysed at the final time with TEM (Figure 7).

### 2.4. The Pharmaco-Chaperones Protect Granular Cells of Mouse Cerebellum from Toxicity of the hIAPP_1–37_ Oligomers

**Figure 8:**
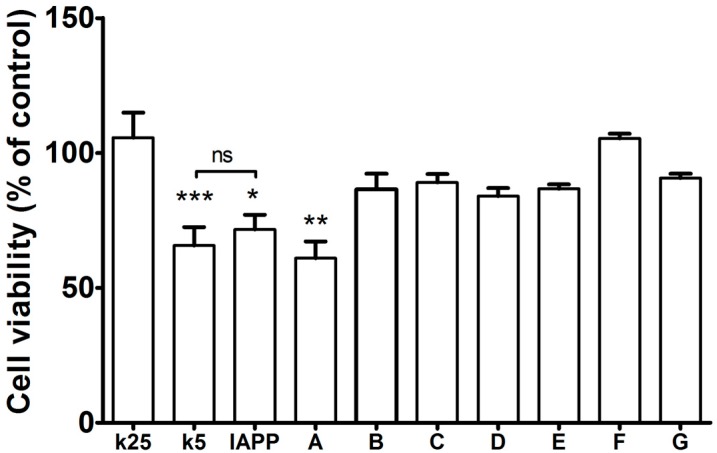
Effect in cells survival at increasing concentrations of hIAPP_1–37_ oligomer: We added increasing concentrations of hIAPP_1–37_ and observed apoptosis statistically different with the dose of 80 µmol/L. We verified that the concentrations of DMSO used to dilute the chaperones did not interfere in the cell viability by itself. We also verified that the dose of the vehicle where the hIAPP_1–37_ was diluted did not interfere in cell viability. CGN cells were treated with 80 μmol/L of hIAPP_1–37_ cytotoxic oligomers plus 40 μmol/L of each chaperone. All conditions, except K5, were incubated with DMSO 0.8%. K25 and K5 are potassium 25 mmol/L (normal) and 5 mmol/L (toxic), respectively as controls; * *p* < 0.05, ** *p* < 0.01, *** *p* < 0.001 when compared with K25 as control. One-way ANOVA and post-hoc Newman-Keuls Multiple Comparison Test. hIAPP_1–37_ cytotoxic oligomers treatment as well as K5 reduces in approximately 50% CGN viability. Chaperones prevent neuron cell death, except for Chaperon **A**.

It is well known that hIAPP_1–37_ can misfold in the pancreas and then migrate through the blood-brain barrier and initiate seeding in the brain [14]. Diabetes mellitus is a strong risk factor for the development of Alzheimer Disease for their overlapping pathophysiological mechanisms such as amyloidogenic events, oxidative stress, and so forth [53]. Hence, we chose cerebellar cells to evaluate in more astringent conditions the pharmaco-chaperone as potential drugs against diabetes and its comorbidities. In order to prove that the chaperones could avoid the toxicity of the hIAPP_1–37_ oligomers, cell viability assays (by MTT) were carried out in cerebellar granule neurons (CGN) (Figure 8). If it protects neurons that are more labile, then it can protect any other cell [53,54,55,56,57].

Several studies have shown that CGN cultured with physiological concentrations of potassium (5 mM KCl; K5) during more than five days in vitro (DIV) die by apoptosis [58,59]. In contrast, the survival and differentiation of these cells in culture are markedly increased in the presence of high concentrations of potassium (25 mM KCl; K25) [59,60]. Thus, culturing CGN with K25 for 7–8 DIV and then transferring the cells to an identical medium with K5, induces apoptotic death of CGN in 24–48 h. It is widely accepted that K5 is a typically pro-apoptotic model and it is a suitable tool for this paradigm [58,59]. In addition, it has been shown that cerebellar granule neurons (CGN) treated with K25 do not show any significant sign of necrotic death. Therefore, in this study the control is the condition that promotes survival (K25) and the experimental condition inducing apoptotic death is K5.

The experimental conditions as dose of hIAPP_1–37_ oligomers and the vehicles used for preparing IAPP (using acetic acid as described by Meier [61]) and chaperones (using DMSO) solutions were evaluated which did not affect the cell viability by itself. All chaperones, except A, maintain the same cell viability as the control under a normal concentration of potassium (K25; not significant differences) and they also reversed the IAPP toxic effect (Figure 8).

## 3. Discussion

The Diabetes mellitus in particular, and the conformational diseases in general, are the medical challenges of the 21st century since they defy the physio-pathological, diagnostic and therapeutic paradigms known to date [1,6,48,62].

**Figure 9:**
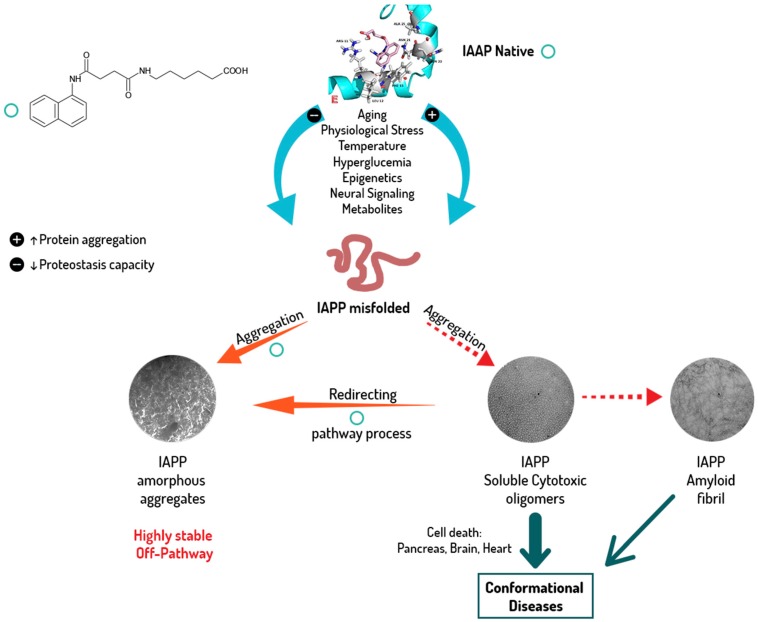
Proposal mechanism of pharmaco-chaperone **E** on protein aggregation/disaggregation. Pharmaco-chaperone **E** can regulate fibre formation processes by binding to the native state, minimizing the formation of IAPP misfolded and cytotoxic oligomers, and in the case of existing IAPP misfolded and cytotoxic oligomers, redirecting the pathway process. Furthermore, it interacts with the amylin fibres and avoids their growth and propagation.

Our strategy to modulate the process of fibrillogenesis was to use pharmaco-chaperones derived from naphthalene (Table 1). Given that Islet Amyloid Polypeptide (hIAPP_1–37_) is the most abundant protein in amyloid fibres in the pancreas and its toxic oligomers are a crucial element in the physiopathology of Diabetes Mellitus, we have studied the hIAPP_1–37_ aggregation-oligomerization pathway (Figure 5, Figure 6, Figure 7, Figure 8 and Figure 9). Using a wide array of methods, strategies and ideas derived from extant knowledge in organic chemistry, protein physio-chemistry, biochemistry, cell biology, and proteomic medicine, we demonstrate that these new naphthalene-derived pharmaco-chaperones help modulate hIAPP_1–37_ aggregation processes (Figure 5, Figure 6, Figure 7, Figure 8 and Figure 9). Approximately, in terms of the reduction of fibre formation as shown by the ThT experiments, the inhibition scale of the pharmaco-chaperones is **E** > **G** > **B** > **A** (Figure 5 and Figure 6) at molar relation protein: Chaperone 1:0.5. Contrary, the chaperone **C** favours the formation of fibres as described by Fortin et al. [35]. Meanwhile, the scale of delay of the fibres formation would be: **E** ~ **G** >**D** > **C** > **B** > **A**. τ lag, “represents a time required for the nuclei that are formed early on in the reaction to grow and proliferate in order to reach an aggregate concentration” [63]. This property can be useful in a chronic stage of diabetes because it stabilizes the native and fibril structures by halting self-catalysis and the creation of cytotoxic oligomers due to fibre formation [28]. At 1:1.5 molar ratio only the chaperone **E** showed a significant decrement in the fibril formation similar to the effect observed at 1:0.5 (Figure 4, Table 1). Chaperone **E** presented the best results, both in the delay and in the decrease of the fibres formation (Figure 5 and Figure 6); this data agrees with the results observed in the TEM micrograph (carried out at 1:0.5 molar ratio), which reveal the absence of fibres (Figure 7). Furthermore, said molar ratio showed that the treatment of hIAPP_1–37_ oligomers with chaperone **E** increases cellular viability monitored by the MTT assay (Figure 8). In what respects of the chaperone **A** case, the TEM micrographs show the presence of scarce short fibres and a larger proportion of circular structures named cytotoxic oligomers, which relates with the decrease of cellular viability (Figure 7 and Figure 8); this data is in accordance with the previous reports, which have proven that the intermediates in fibre formation, called cytotoxic oligomers, are responsible for the cytotoxicity in cells [64,65]. The cell damage mechanism that has been proposed is such since oligomers are capable of binding to cellular membranes to allow the formation of pores, which would lead to the loss of selectiveness in membranes, followed by the activation of molecules implicated in cellular death [66]. Meanwhile, the TEM micrographs of the presence of chaperones **B**, **D** and **F**, showed a large quantity of fibres but no presence of cytotoxic oligomers, fact that is in agreement with the cell viability assays (Figure 7 and Figure 8). The results show that most pharmaco-chaperones have a cytoprotective effect; the observations of TEM’s microscopy were that the fibres of the hIAPP_1–37,_ in absence of the chaperones are long, thick and abundant; whereas in presence of the chaperones they are short, thin and in minor quantity, or in some cases absent (Figure 7 and Figure 8). We have several multi-target effects while Jiang and collaborators only have the cytoprotective effect [67], or in the case of BRICHOS, only stabilize the fibril structures halting self-catalysis [14]. These results are promising in the research of compounds that inhibit or delay the fibril formation process or in addition work as an “off-pathway” drug (Figure 3, Figure 4, Figure 5, Figure 6 and Figure 7).

According to the molecular docking studies, the chaperones interact with the region comprised between amino acids 11 to 28, by hydrogen bonds, hydrophobic interactions, and by Van der Waals forces. This region is one of those reported by Eisenberg et al. [36,37], which is involved in the formation of the steric zipper. All chaperones engage strongly through the interactions π∙∙∙π stacking with key amino acid Phe15. In addition, hydrogen bonds with Arg11 favours native state of the peptide, this suggests that these molecules could prevent the formation of hIAPP_1–37_ dimer. The aforementioned interactions, in particular the chaperone **E**, have three probable additional hydrogen bonds (Arg11, Asn14 and Asn31) which should contribute to a greater stability of the native species, blocking the potential sites of peptide dimerization. These in silico results are in concordance with those found in vitro and could be the explanation by which chaperone **E** delays and decreases the amount of cytotoxic oligomers formed. The challenge was to find a pharmaco-chaperone that would be able to interact with the native form and the amyloid fibres of the amylin. Chaperone **E** meets this double function since, as shown by this research, in solution form it interacts with the native form of hIAPP_1–37_ (Figure 2 and Figure 5), and avoids fibrillar growth and propagation (Figure 3). Comparing chaperone **E** with curcumin (an anti-amyloid drug) we can see that the first one has more interaction surface, hence, being much more effective in the inhibition of the fibre formation and its propagation. As a matter of fact, curcumin, as seen by the docking studies, is significantly similar to chaperone A in the ligand-protein interaction (Figure 2, Figure 3 and Figure 4).

The modulation of proteostasis by the family of pharmaco-chaperones **A**–**F** is a promising potential approach to limit the onset and progression of diabetes and its comorbidities (Figure 9). As a consequence we got additional insights into the complex mechanisms of protein aggregation and its effects on conformational diseases (Figure 9).

The bonding sites construct structural blocks that can overlap in various target proteins and make a meta-structure called meta-pharmacophore, which allows the development of novel drugs. Our results are novel and highly promising, for they allow researchers to build a set of molecules that can be used for studying and potentially treating the various physio-pathological stages of diabetes. For instance, when dealing with an acute phase of cytotoxicity, what is needed is the recruitment of cytotoxic oligomers, thus having chaperones that accelerate fibre formation would be very useful, whereas in a chronic stage it is better to have a chaperone that stabilizes the native and fibril structures in order to halt self-catalysis and the creation of cytotoxic oligomers as a consequence of fibre formation. This is demonstrated by means of analyses of the apoptosis produced by hIAPP_1–37_ toxic oligomers, which results in a protective effect of many chaperones regardless of their capacity for accelerating or inhibiting in vitro formation of fibres. This is extremely relevant as it allows for a close examination of the fibre-formation phenomena and, potentially, to control cytotoxicity. The pharmaco-chaperons act as modulators that provide dynamic interventions and the multi-target capacity (native state, cytotoxic oligomers, protofilaments and fibrils of hIAPP_1–37_) required to meet the treatment challenges of diabetes.

## 4. Materials and Methods

### 4.1. Preparation and Characterization of Chaperones and hIAPP_1–37_

Chaperones **A**, **B**, **C**, **D**, **E**, **F** and **G** were synthesized and purified at Neuroscience Centre of Cuba (Havana, Cuba) (see Table 1) [68]. Chaperones were dissolved in DMSO at stock concentrations of 50 mM. hIAPP_1–37_ was purchased from ProteoGenix, France. Lyophilized hIAPP_1–37_ was dissolved in DMSO to prepare a 256 µmol/L stock solution, aliquots were stored at −80 °C. The stocks were then diluted with the buffer to obtain the desired final hIAPP_1–37_ or chaperone concentrations just before use.

### 4.2. Oligomer Preparation

hIAPP_1–37_ oligomers were prepared essentially as described by Kayed [64]. Briefly, 1 mg of hIAPP_1–37_ was solubilized in 400 μL of hexafluoro-2-propanol (HFIP) for 15 min at room temperature. 100 μL of the resulting hIAPP_1–37_ solution were added to 900 μL Mili-Q H_2_O in a siliconized Eppendorf tube. After 20 min of incubation at room temperature the samples were centrifuged for 15 min at 14,000× *g* at 4 °C and the supernatant fraction (pH 2.8–3.5) was transferred to a new siliconized tube and subjected to a gentle stream of N_2_ for 10 min to evaporate the HFIP. The samples were then stirred at 500 rpm using a heating block from Thermomixer comfort Eppendorf for 30 min at 22 °C. 10 μL aliquots were taken for observation by Transmission Electron Microscopy (TEM). In order to prepare highly pure samples, residual trifluoroacetic acid was removed by lyophilisation in 0.1 M HCl followed by lyophilisation in 50% acetonitrile. Then, the lyophilized oligomers of hIAPP_1–37_ were dissolved in DMSO to prepare a 256 µmol/L stock solution. The stock solution was then diluted with the medium to obtain the desired final hIAPP_1–37_ concentrations.

### 4.3. Molecular Docking

The crystallographic structure of hIAPP_1–37_ was downloaded from Protein Data Bank (PDB, entry 2L86). The structures of the chemical chaperones were refined using Avogadro software and converted to PDBqt (Protein Data Bank in format qt) with the program Autodock Tools, considering all ligand bonds as flexible. The chaperones were docked with hIAPP_1–37_ using AutoDock Vina [69]. The determination of the interaction zones between the IAPP amino acids and all of the tested compounds were carried out using the molecular graphic program UCSF Chimera [70]. All calculations were performed on a cluster of 10 computers (30 CPU) with Linux as operating system.

In addition, the Amylin fibrils of 4 monomers in size (amylin_single-1.pdb) was downloaded from fibrilizer-a computational tool to build polymorphic amyloid fibrils at an atomic level resolution: http://amyloid.cs.mcgill.ca/database/Amylin/Amylin.html#single [40,41,42]. The chaperones **A**, **E** and curcumin as positive control were docked with amylin fibrils using AutoDock Vina. The rest of the analysis was made as above.

### 4.4. Thioflavin T (ThT) Fluorescence Assay

ThT Fluorescence assay was performed as described by Meier [61] and Peinado [71]. Briefly, hIAPP_1–37_ fibril formation in the presence or absence of chaperones **A**, **B**, **C**, **D**, **E**, **F** and **G**, was monitored using thioflavin T (ThT) fluorescence, a dye known to preferentially bind amyloid fibrils as previously described [72]. All the experiments were performed in triplicate at 20 °C. ThT fluorescence increases in a solution of freshly reconstituted hIAPP_1–37_ as amyloid fibrils grow. The samples of hIAPP_1–37_ at a concentration of 12.8 µmol/L were incubated in the presence of chaperone **A**, **B**, **C**, **D**, **E**, **F** and **G**, at a molar ratio of hIAPP_1–37_/chaperone of 1:0.5, 1:1, and 1:1.5 in 20 mM Tris buffer of pH 7.4 with a 100 mmol/L of NaCl. Each fibril formation reaction was performed and real-time emission intensities were measured at 482 nm with excitation at 450 nm. Measurements were performed at room temperature (20 °C) with excitation and emission slit widths of 1 and 10 nm, respectively. Fluorescence measurements were registered using M1000 Tecan (Viena, Austria). Plots of ThT emission intensity as a function over the time were fitted to a sigmoidal curve (nonlinear regression analysis) using Origin Software.

### 4.5. Transmission Electron Microscopy

A 6 μL droplet of the fibrillization reaction was deposited on a 400-mesh copper grid coated with collodion film, and allowed to settle for 4 min. The excess solution was wicked away by gently applying a piece of blotting paper to the edge of the grid. Then a 40 μL droplet of 2% uranyl acetate was deposited on the grid and allowed to settle for 60 s. The excess solution was removed as mentioned above. The grid was left air-drying for 24 h to be later observed using a JEM-1010, JEOL (Tokyo, Japan) microscope operated at an acceleration voltage of 80 kV. The electronic micrographs were acquired using a MTI model CCD-300-RC camera (Tokyo, Japan). All the experiments were performed at least three times

### 4.6. Primary Cell Cultures

Cerebellar granule neurons (CGN) were prepared as previously described [73]. Briefly, cell suspensions dissociated from 8-day-old Wistar rat cerebellum were plated at a density of 1.5 × 10^5^ cells/cm^2^ in dishes coated with poly-L-lysine (5 mg/mL). Basal Medium Eagle (BME) was supplemented with 10% (*v*/*v*) heat inactivated foetal calf serum, 2 mM glutamine, 4.5 mM glucose, 20 mM KCl, 50 U/mL penicillin and 50 mg/mL streptomycin. The dishes were incubated at 37 °C in a humidified 5% CO_2_/95% air atmosphere. Cytosine arabinoside (10 µmmol/L) was added 20 h after seeding. Cells were maintained for 6–8 days in vitro (DIV).

All the animals were obtained from the Vivarium of the Cell Physiology Institute, University of Mexico. Wistar rat pups of 6 to 8 postnatal days were euthanized by decapitation following the recommended procedures to avoid unnecessary infliction of pain and using the strictly necessary animals.

All animal studies were performed in compliance with UNAM Animal Care Guideline NOM-062-ZOO 1999. The study protocol was approved by the Ethical Committee of Instituto de Fisiología Celular, UNAM. SAGARPA-SENASICA AUTO-B-C-1216-030

### 4.7. Cell Viability

Reduction of MTT (3-[4,5-dimethylthiazol-2-yl]-2,5-diphenyl tetrazolium bromide) was used to assess cell viability as described previously [74,75,76]. To test the effects of chaperones or oligomers, cells were seeded on a 48 well plate at 1.5 × 10^5^ cells/mL. The next day, the medium was replaced with fresh medium containing freshly dissolved hIAPP_1–37_ (80 µmol/L), chaperones (40 µmol/L) molar ratio of hIAPP_1–37_/chaperone of 1:0.5, or vehicle (acetic acid 0.002%) for hIAPP_1–37_ as described by Meier [61] and DMSO 0.8% for chaperones as described by Sablón [28]. Twenty-four hours later, MTT (0.1 mg/mL) was added to the CGN and incubated for 15 min at 37 °C. After the removal of medium containing MTT, DMSO 100% was added to the dishes and incubated during 15 min at room temperature in darkness. Formazan blue formed was measured at 560 nm in an ELISA plate reader (microplates ELx800, BioTek, Winooski, VT, USA). Values were corrected with the background signal. All the experiments were performed at least three times.

### 4.8. Statistical Analysis

Data are presented as mean ± SM. Statistical Analyses were carried out by ANOVA followed by Fisher’s test for Vmax analysis and Tukey test for tau analysis using GraphPad PRISMA 7.02. Statistical significance was determined using Newman–Keuls Multiple Comparison Test. A *p* value ≤ 0.05 was considered significant.

## 5. Conclusions

We used a cross-functional approach that combines in silico and in vitro biochemical and biophysical methods to study the hIAPP_1–37_ aggregation-oligomerization process as to reveal novel potential anti-diabetic drugs. The family of pharmaco-chaperones are modulators of the oligomerization and fibre formation of hIAPP_1–37_ by interacting with the amino acid in the amyloid-like steric zipper zone. They inhibit and/or delay the aggregation-oligomerization pathway by binding and stabilizing several amyloid structures of hIAPP_1–37_. Furthermore, they are able to protect cerebellar granule cells (CGC) from the cytotoxicity produced by the hIAPP_1–37_ oligomers. Our results provide evidence that the pharmaco-chaperones may be a fruitful approach to delay the onset and progression of diabetes.

## Figures and Tables

**Table 1:** Values of ν max and τ lag in IAPP_1–37_ aggregation with the incubation of chaperones.

Condition	Dose (µM)	Vmax	Sd	ν max (%)	τ lag	Sd	τ lag (%)
IAPP_1–37_	12.8	6944	772	100	3.989	0.37388	100
Chaperone **A** 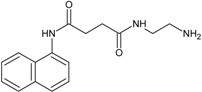	6.4	6701	775	97	4.172	0.42174	105
	12.8	9509	1032	137	3.973	0.35464	100
	64	6582	730	95	3.967	0.372	99
Chaperone **B** 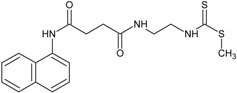	6.4	5783	707	83	4.250	0.46283	107
	12.8	8562	1100	123	4.602	0.55049	115
	64	5328	672	77	4.639	0.51981	116
Chaperone **C** 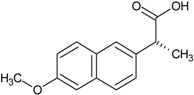	6.4	9128	1217	131	4.650	0.60413	117
	12.8	8791	1175	127	4.797	0.6116	120
	64	6348	885	91	4.840	0.65495	121
Chaperone **D** 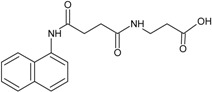	6.4	6946	1126	100	5.787	1.14215	145
	12.8	6326	944	91	5.212	0.80756	131
	64	5255	289	76	2.071	0.24877	52
Chaperone **E** 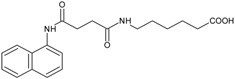	6.4	4341	783	63	6.320	1.48342	158
	12.8	9055	1488	130	5.559	1.0796	139
	64	4465	835	64	6.569	1.76324	165
Chaperone **G** 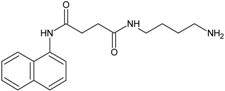	6.4	5182	867	75	6.322	1.36815	158
	12.8	8455	1281	122	5.294	0.86324	133
	64	6252	847	90	5.659	0.81244	142

**Table 2:** In silico determination of contact percent of the pharmaco-chaperones with the amino acids of IAPP_1–37_ involved in the interaction.

	Contact % with aa of hIAPP_1–37_										
	Cys7	Arg11	Leu 12	Asn 14	Phe 15	His 18	Asn 21	Asn 22	Ala 25	Ser 28	Asn 31
Chaperone **A**	-	90	92	91	89	88	85	90	90	91	-
Chaperone **B**	-	91	89	91	90	90	80	-	-	89	-
Chaperone **C**	-	89	90	91	89	90	82	91	92	90	-
Chaperone **D**	-	87	91	91	87	88	85	88	88	91	30
Chaperone **E**	-	96	98	96	97	95	98	95	98	98	92
Chaperone **F**	-	90	92	88	84	80	86	87	89	90	-
Chaperone **G**	50	90	92	87	91	90	87	90	90	89	40
